# Genomic Comparison of *Agrobacterium pusense* Strains Isolated from Bean Nodules

**DOI:** 10.3389/fmicb.2016.01720

**Published:** 2016-10-27

**Authors:** Alejandro Aguilar, Humberto Peralta, Yolanda Mora, Rafael Díaz, Carmen Vargas-Lagunas, Lourdes Girard, Jaime Mora

**Affiliations:** Programa de Genómica Funcional de Procariotes, Centro de Ciencias Genómicas, Universidad Nacional Autónoma de MéxicoCuernavaca, Mexico

**Keywords:** *Agrobacterium*, symbiosis, *Phaseolus vulgaris*, nodulation

## Background

Rhizobia are soil bacteria that can interact with legumes through the formation of root nodules, where they fix nitrogen symbiotically. Rhizobia are also present in other legume tissues, including the interiors of roots and leaves, as well as in other plants and trees (Sturz et al., 1997; López-López et al., 2010; Rozahon et al., 2014). We recently reported the isolation and characterization of strains from common bean seeds (*Phaseolus vulgaris*) capable of nodulating and fixing nitrogen when inoculated onto bean (Mora et al., 2014). The seed-borne strain CCGM7 was characterized by screening for genes that allow the bacteria to survive in seeds (Peralta et al., 2016). To extend this research, we tested nodules formed by CCGM7 (or a strain from soil, CFNEI73) and isolated two strains, named CCGM10 and CCGM11, capable of growing in LB medium, a phenotype reported for certain strains of agrobacteria (Tanaka et al., 2009).

Genus *Agrobacterium* includes several species of bacteria commonly found in soil capable of forming tumors on the stems and roots of plants and trees (Singh and Prasad, 2016). Consistent with this phenotype, introduction of *Agrobacterium* T-DNA into the plant genome results in tumor formation (Bourras et al., 2015). *Agrobacterium fabrum* (formerly *A. tumefaciens*) has been widely used as a natural genetic engineer to transform plants of agricultural interest (Lassalle et al., 2011); however, *A. radiobacter* does not form tumors.

The newly defined species *Agrobacterium pusense* includes the type strain NRCPB10^T^, isolated from *Cicer arietinum* roots (Panday et al., 2011; Mousavi et al., 2015), as well as other root and nodule bacteria generally incapable of forming nodules or fixing nitrogen. Strain H13-3 has been studied for many years as a model for chemotaxis and motility, but it is non-symbiotic and was isolated from the rhizosphere of *Lupinus luteus* (Wibberg et al., 2011). Strain HPC(L) was isolated from desert soil (Agarwal and Purohit, 2013). Other strains closely related to *A. fabrum* have been found in nodules of legumes, including common bean, but cannot fix nitrogen (De Lajudie et al., 1999; Mhamdi et al., 2002, 2005; Aserse et al., 2012). IRBG74, the only strain with a symbiotic plasmid, can fix nitrogen in *Sesbania cannabina* and infect rice endophytically (Tan et al., 2001; Crook et al., 2013). Intriguingly, several isolates of *A. pusense* have been found in human wounds and body fluids (Aujoulat et al., 2015). Although genus *Agrobacterium* is to some extent phylogenetically entangled with rhizobia (see a recent review on *Rhizobium* taxonomy, Mousavi et al., 2015), one means for clear identification is the linear chromosome present in several *Agrobacterium* strains, which is accompanied by a protelomerase gene (*telA*) (Ramírez-Bahena et al., 2014).

Here, we report the genomic sequences of the *A. pusense* strains CCGM10 and CCGM11 isolated from bean nodules, a genomic analysis of the homology of their chromosomes and plasmids relative to other agrobacteria, and a functional profile of their genes.

## Methods

### Strains and culture media

Strains were maintained on solid rich medium LB (1% peptone, 0.5% yeast extract, 1% NaCl), supplemented with appropriate antibiotics. Liquid cultures for inoculation were grown in LB at 30°C with shaking at 200 rpm (Encarnación et al., 1995). Antibiotics were added as follows: nalidixic acid, 20 μg/mL; streptomycin, 200 μg/mL; and fosfomycin, 200 μg/mL.

### Isolation of *A. pusense* strains CCGM10 and CCGM11

*P. vulgaris* Negro Jamapa plantlets were inoculated with *Sinorhizobium americanum* strain CFNEI73 or CCGM7, both of which form nodules and fix nitrogen (Toledo et al., 2003; Mora et al., 2014). When the nodules were crushed and applied to LB plates, where symbiotic rhizobia normally do not grow, single colonies appeared after 72 h of incubation at 29°C. Three colonies from each spot were washed exhaustively in 10 mM MgSO_4_/0.01% Tween 40 and plated on LB medium, followed by incubation for 72 h at 29°C. To purify the strains, bacteria were grown overnight in liquid LB, serially diluted in 10 mM MgSO_4_-0.01% Tween 40, plated on LB plates, and incubated as above. Individual colonies were isolated from these plates.

### Visualization of plasmids

Plasmids were visualized by the Eckhardt technique with modifications, as previously reported (Hynes and McGregor, 1990).

### Genome sequencing, assembly, and annotation

Total DNA from strains was extracted from cultures grown in liquid LB medium; the cells were centrifuged, resuspended in 50 mM Tris/20 mM EDTA pH 8.0, and then lysed with proteinase K (2.5 mg/mL) and 10% SDS. After treatment with a mix of 1:1 phenol-chloroform, DNA was precipitated with absolute ethanol. Genomes were sequenced by Macrogen (Seoul, South Korea). For each strain, libraries of 3 kilobase pairs (kb) were prepared and run on an Illumina HiSeq2000 to obtain 100–base pair (bp) mated pair reads. Total read counts were 13,969,230 and 17,424,244 for CCGM10 and CCGM11, respectively; filtered read counts were 6,951,885 and 8,690,792, with genome coverage of 121 × and 152 ×. Assembly was performed with SOAPdenovo2 (Luo et al., 2012). Annotation was performed with RAST v4.0 (Aziz et al., 2008) with manual curation. Functions were assigned using the extended annotation of clusters of orthologous groups (COG) of COG 2014 database update (Galperin et al., 2015). Phages were detected using the PHASTER server (Arndt et al., 2016).

### Comparative genomic analysis

The *rpoB* genes of strains CCGM10 and CCGM11 were sent to BLAST server (http://www.ncbi.nlm.nih.gov/Blast.cgi) to detect closely related strains. The genomes of selected strains were compared with genomes of CCGM10 and CCGM11 using Proteinortho version 5, with BLAST parameters E < 1E-5; 30% identity, and 70% coverage. The organisms selected for comparison were *A. pusense* IRBG74; *A. pusense* HPC(L), also known as *R. pusense*; and *A. fabrum* C58, also designated *A. tumefaciens*.

### Nucleotide accession numbers

The genomes of *A. pusense* strains CCGM10 and CCGM11 were registered at GenBank with the provisional accessions LNZW00000000 and MAPG00000000, respectively.

## Results

### Homology and chromosomal conservation

**Figure 1:**
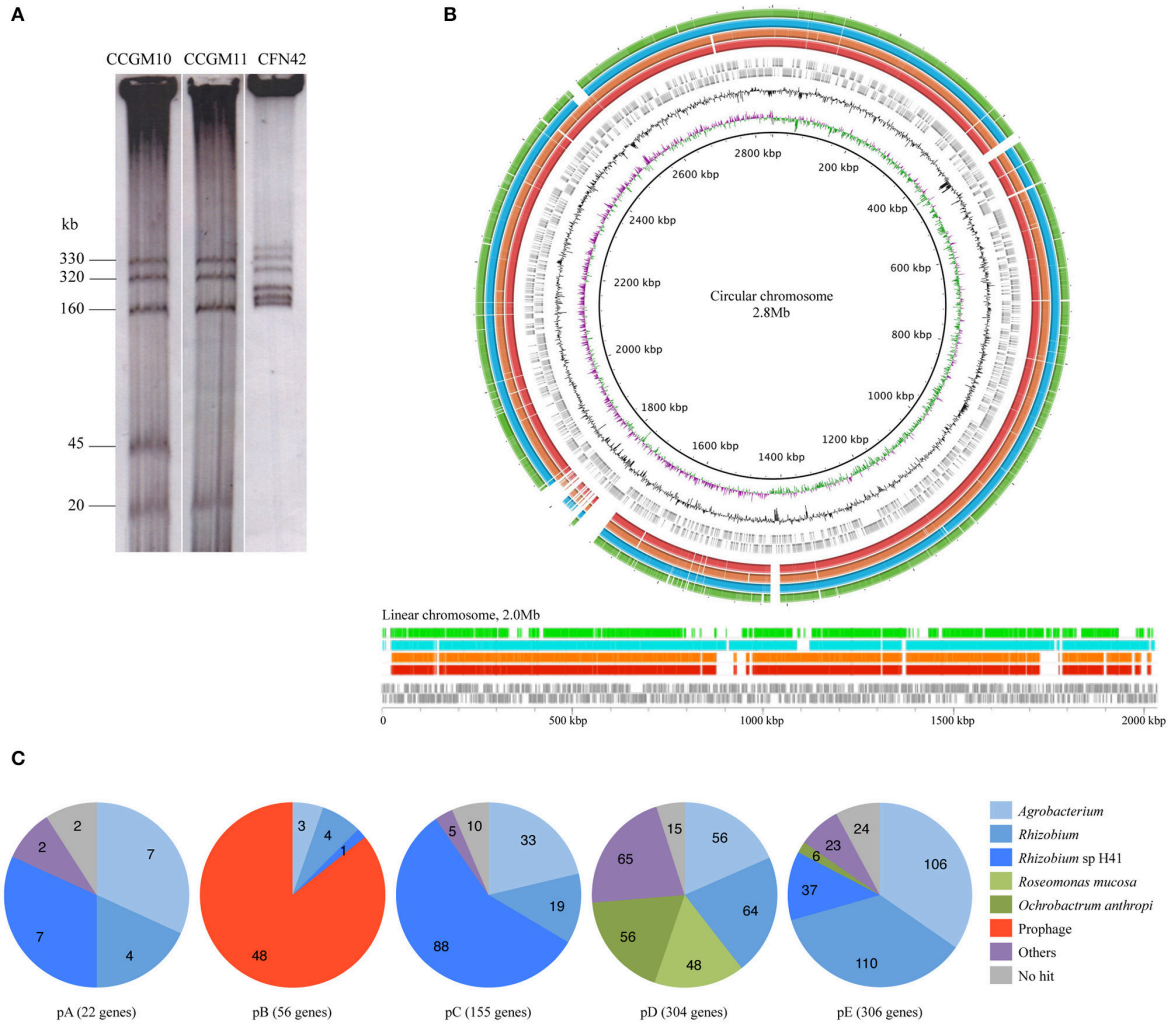
**Genome comparison of ***Agrobacterium pusense*** strains**. **(A)** Eckhardt plasmid profile of strains CCGM10, CCGM11, and *R. etli* CFN42 (for which the pattern was known). Estimated molecular weights are in kb. **(B)** Schematic representation of the chromosomes of *A. pusense* strains. The chromosomes of *A. pusense* IRBG74 were used as a reference. Top, comparison of circular chromosome. From the innermost circle: GC skew, %GC content, CDS prediction with direction of transcription of the IRBG74 chromosome (gray boxes), comparison with CCGM10 (red bars), comparison with CCGM11 (orange), comparison with HPC(L) (turquoise), and comparison with *A. fabrum* C58 (green). Bottom, comparison of linear chromosome. From bottom, CDS prediction with direction of transcription of the IRBG74 chromosome (gray boxes), comparison with CCGM10 (red bars), comparison with CCGM11 (orange), comparison with HPC(L) (turquoise), and comparison with *A. fabrum* C58 (green). **(C)** Homology of plasmid genes of strain CCGM10.

The main characteristics of the genomes of *A. pusense* strains CCGM10 and CCGM11 are listed in Table 1. The similarity between the strains was remarkable, with 93 and 92% of shared orthologs in the circular and linear chromosomes, respectively (Supplementary Table 1). Interestingly, some portions of the chromosomes were not shared with strain IRBG74; these segments mainly corresponded to genes with hypothetical functions (Supplementary Table 2). The sequence identity of shared orthologs in both strains was surprisingly high, 99.97%, and 5096 products had 100% identity at the amino acid level. Only about 142–205 genes in each chromosome were strain-specific. In addition, the plasmids were almost the same size (Figure 1A) and exhibited very high proportion of shared orthologs, ranging from 82 to 95%. These features indicate that the strains diverged very recently, mainly by insertion or deletion of genes.

**Table 1:** **Genomic features of ***A. pusense*** strains**.

**Feature**	**CCGM10**	**CCGM11**
Genome size (Kb)	5802	5755
No. of scaffolds	35	31
Coverage	93x	125x
N50 (bp)	978,610	982,548
No. of CDS	5787	5693
No. of tRNAs	49	53
%G+C content	59.3	59.3
CDS per replicon:		
Circular chromosome	2934	2923
Linear chromosome	1955	1949
Plasmid A	22	22
Plasmid B	56	–
Plasmid C	155	155
Plasmid D	304	297
Plasmid E	306	296
Not assigned	55	51

*CDS, coding sequence*.

A structural analysis of the genomes of these strains revealed very high conservation of both the linear and circular chromosomes, as shown in Figure 1B. The genomes were compared with that of the strains' closest relative, *A. pusense* IRBG74. The chromosomes were highly conserved, but the symbiotic plasmid of strain IRBG74 was not shared (not shown), and some regions were exclusive to the chromosomes of the reference strain. The genomes of *A. pusense* HPC(L) and that of *A. fabrum* C58, the model organism of the genus, were also included in the comparison.

### Homologs of plasmid genes

Homology analysis of plasmid genes revealed that many had homologs in rhizobia and agrobacteria, but also with more distant organisms (Figure 1C). Most of genes in plasmid E were related to those from *Agrobacterium* and *Rhizobium*. Plasmid D harbored numerous genes with homologs in *Roseomonas mucosa* (with relatives isolated from human blood; Han et al., 2003) and *Ochrobactrum anthropi*, a member of the Brucellaceae that is found in natural ecosystems but also able to infect humans (Daxboeck et al., 2002). More than half of the genes on plasmid C were related to those from *Rhizobium* sp. H41, isolated from eroding rocks (Xi et al., 2014). Plasmid B, which is absent from strain CCGM11, is an intact temperate prophage of about 37 kb, harboring 47 genes, some shared with a prophage present in the circular chromosome of *A. tumefaciens* C58 and related to phage AmM-1 from *Aurantimonas* sp., a psychrotolerant bacterium belonging to the order Rhizobiales found in the deep ocean (Goldstein et al., 1982; Yoshida et al., 2015). Some genes assigned to plasmid A had homologs in *Rhizobium* sp. H41.

### Other prophages in the genome

In light of our detection of the prophage in plasmid B, we analyzed the remaining replicons and identified additional intact prophages (Supplementary Table 3). The circular chromosome of strains CCGM10 and CCGM11 harbored three prophages: two related to phages RR1 and 16-3, also present in rhizobia, and vB_PmaS from the marine bacterium *Paracoccus marcusii*. In the putative linear chromosome of both strains, we found an intact prophage related to RC1 from *Rhodobacter capsulatus*. All prophages belonged to the family Caudovirales.

### Gene functions

Next, we analyzed the functions of genes in the plasmids of strain CCGM10 (Table 1 and Supplementary Table 4). Plasmid E was enriched in genes encoding transcriptional regulators and factors involved in signal transduction and amino acid or ion transport. Among these genes were those encoding proteins related to DNA uptake and folding (as HU), *dnaE* and *ligD*; central metabolism (e.g., *fabG, ilvB*, and *arcCD*), and ubiquinone synthesis. Plasmid D was especially enriched with genes involved in transcription, replication, and intracellular trafficking. Other notable genes encoded factors associated with secretion system IV, chemotaxis (*cheBRW* and methyl-accepting proteins *mcp*), transcriptional regulators and sensors, and genes for the redox response involving flavodoxin and glutathione. In addition, the plasmid contained a 500 bp segment homologous to the T-DNA segment of plasmid pTi.

Plasmid C was rich in genes involved in trafficking and ion transport, and it also harbored genes related to defense (toxin-antitoxin pairs) and central metabolism, such as *arcACD, argF, speD*, and the universal stress protein *uspA*. Plasmid A contained some genes involved in chemotaxis and glycerol utilization. In addition, we identified clusters of genes involved in conjugal plasmid transfer and replication (*repABC* family) in plasmids pC, pD, and pE.

In the circular chromosome, we identified the following genes involved in nitrogen fixation: *fixGHS, fixNOQP, nifURS, nodLN*, and *fixR*. *A. fabrum* C58 harbored homologs of these genes. However, some other genes not present in the C58 genome, such as the *fixLJK* cluster, were present in plasmid D. FixLJ are transcriptional regulators of *nifA*, the general activator of nitrogen fixation; FixK is regulator of the *fixNOQP* operon (encoding Cbb3, the cytochrome used by symbiotic rhizobia for respiration inside the nodules). These genes may represent the remnant of a complete gene cluster related to nitrogen fixation.

### Bean seeds as the origin of *A. pusense* strains

Several clues point to bean seeds as the origin of this pair of *A. pusense* strains, which were isolated from nodules but lack genes for nodulation. First, we have isolated 10 other rhizobial strains from nodules of non-inoculated plants; seeds from plants inoculated with two selected strains, CCGM1 or CCGM7, yielded positive signals for specific rhizobial gene markers (Mora et al., 2014). Second, some of the seed rhizobia strains did not have nodulation genes, but they are nonetheless able to inhabit nodules. Third, seed rhizobia have been identified as pairs of highly related strains differing in only one plasmid, as in this *A. pusense* pair. No *A. pusense* strains have been used previously in our greenhouse. It is possible that the *A. pusense* strains originally harbored a symbiotic plasmid that has been lost, but is still present in close relatives such as IRBG74. As with other agrobacteria, it will be of interest to define the precise symbiotic role of our isolates (Mrabet et al., 2006).

## Availability of data

The files containing the genomic sequences and the data resulting from our analyses are available and can be accessed freely at our server by ftp (ftp://kanan.ccg.unam.mx/PGFP/Apusense). A README file explains the content and mode of use.

## Author contributions

JM conceived and coordinated the project. AA performed assembly and annotation of the genomes. AA and HP performed the homology and functional analyses. YM, RD, CV, and LG performed the isolation and identification of the strains. HP and JM drafted the manuscript. All authors have read and approved the manuscript.

## Funding

This project was partially supported by grants from the Consejo Nacional de Ciencia y Tecnología–Mexico (213606 and 152776) and from DGAPA-PAPIIT-UNAM (IN208216 and IN206914). The agencies had no role in the design of the study or the analysis or interpretation of the results.

### Conflict of interest statement

The authors declare that the research was conducted in the absence of any commercial or financial relationships that could be construed as a potential conflict of interest.
