# Supplementary material for: Genomic Comparison of Agrobacterium pusense Strains Isolated from Bean Nodules
Source: Front Microbiol. 2016 Oct 27;7:1720. doi: 10.3389/fmicb.2016.01720 (PMC5081363; doi:10.3389/fmicb.2016.01720)
Supplement: Supplementary file 1 [file Table1.PDF]

Supplementary table 1. Orthologs between strains CCGM10 and CCGM11.

Replicon	No. of CDS		Shared orthologs	Strain-specific genes	
	CCGM10	CCGM11		CCGM10	CCGM11
Circular chromosome	2934	2923	2731	203	192
Linear chromosome	1955	1949	1800	147	142
Plasmid E	306	296	259	46	35
Plasmid D	304	297	247	57	50
Plasmid C	155	155	142	11	11
Plasmid B	56	-	-	56	-
Plasmid A	22	22	21	1	1
Not assigned	55	51	45	5	3
Total	5787	5693	5245	526	434

Strains had other clusters of orthologs containing additionally at least one paralog: 16 for CCGM10 and 14 for CCGM11.
