# Supplementary material for: Genomic Comparison of Agrobacterium pusense Strains Isolated from Bean Nodules
Source: Front Microbiol. 2016 Oct 27;7:1720. doi: 10.3389/fmicb.2016.01720 (PMC5081363; doi:10.3389/fmicb.2016.01720)
Supplement: Supplementary file 2 [file Table2.PDF]

Supplementary table 2. Chromosomal genes of strain IRBG74 located in segments not shared with strains CCGM10 and CCGM11.

Circular chromosome						
**Position**	**Strand**	**Length AA**	**Product_accession**	**Locus_tag**	**Function**	**COG**
47262..48227	+	321	WP_022555329.1	BN877_RS00230	hypothetical protein	COG0741M
112566..112814	-	82	WP_048902633.1	BN877_RS00515	hypothetical protein	-
112978..113361	-	127	WP_048902634.1	BN877_RS00520	hypothetical protein	-
246073..246546	-	157	WP_022555454.1	BN877_RS01170	hemoglobin	COG2346P
381130..381957	+	275	WP_022555540.1	BN877_RS01855	transposase	COG2801X
430383..431576	+	397	WP_022555568.1	BN877_RS02080	integrase family protein	COG0582LX
432000..435260	+	1086	WP_048902648.1	BN877_RS02085	hypothetical protein	-
435257..435718	+	153	WP_022555570.1	BN877_RS02090	response regulator receiver protein	COG0745TK
435724..436947	+	407	WP_022555571.1	BN877_RS02095	hypothetical protein	COG0784T
439221..441425	-	734	WP_022555574.1	BN877_RS02115	hypothetical protein	-
441548..442051	+	167	WP_022555575.1	BN877_RS02120	hypothetical protein	-
442433..442720	-	95	WP_022555576.1	BN877_RS02125	hypothetical protein	-
442798..443835	-	345	WP_048902649.1	BN877_RS02130	hypothetical protein	-
444585..445580	-	331	WP_022555579.1	BN877_RS02135	hypothetical protein	-
478340..479353	+	337	WP_048902652.1	BN877_RS02285	hypothetical protein	-
507716..507943	-	75	WP_004439907.1	BN877_RS02415	DNA gyrase inhibitor YacG	COG3024L
562786..563874	+	362	WP_004440053.1	BN877_RS02720	sugar ABC transporter ATP-binding protein	COG3842E
579470..580567	+	365	WP_004432613.1	BN877_RS02790	putrescine ABC transporter substrate-binding	COG0687E
630131..631081	-	316	WP_004440144.1	BN877_RS02990	LysR family transcriptional regulator	COG0583K
684668..686020	+	450	WP_004440238.1	BN877_RS03245	diguanylate cyclase	COG5001T
686029..686217	+	62	WP_048902658.1	BN877_RS03250	hypothetical protein	-
701864..702676	-	270	WP_022555708.1	BN877_RS03335	hypothetical protein	COG0115EH
702753..703127	+	124	WP_022555709.1	BN877_RS03340	MerR family transcriptional regulatory	COG0789K
793793..793882	+	29	WP_034495918.1	BN877_RS03810	hypothetical protein	-
802757..803122	-	121	WP_022555773.1	BN877_RS03855	conserved exported protein of unknown	COG4190K
803119..803400	-	93	WP_022555774.1	BN877_RS03860	hypothetical protein	-
931754..935557	+	1267	WP_022555866.1	BN877_RS04555	conserved exported protein of unknown	-
987548..987793	+	81	WP_022555893.1	BN877_RS04800	Aminoglycoside 3\'-phosphotransferase	-
989478..989675	-	65	WP_048902669.1	BN877_RS04810	hypothetical protein	-
1040080..1040388	-	102	WP_048902798.1	BN877_RS05055	hypothetical protein	COG2852L
1134395..1134583	+	62	WP_022555982.1	BN877_RS05505	hypothetical protein	-
1313546..1313914	-	122	WP_048902679.1	BN877_RS06390	hypothetical protein	-
1348146..1348469	+	107	WP_006697555.1	BN877_RS06545	hypothetical protein	-
1348731..1349072	-	113	WP_006697554.1	BN877_RS06550	hypothetical protein	-
1349345..1349821	-	158	WP_022556095.1	BN877_RS06555	RHS protein	-
1381051..1383255	+	734	WP_034496207.1	BN877_RS06705	nitrogen fixation protein FixI	COG2608P
1386706..1389171	-	821	WP_048902681.1	BN877_RS06730	beta-mannosidase	COG3250G
1410662..1411963	+	433	WP_022556131.1	BN877_RS06840	hypothetical protein	COG0582LX
1412008..1412907	-	299	WP_022556132.1	BN877_RS06845	hypothetical protein	-
1412907..1414055	-	382	WP_048902682.1	BN877_RS06850	hypothetical protein	-
1416072..1417832	-	586	WP_048902811.1	BN877_RS06865	hypothetical protein	-
1417917..1418180	-	87	WP_048902812.1	BN877_RS06870	hypothetical protein	-
1418491..1418832	-	113	WP_048902683.1	BN877_RS06875	hypothetical protein	-
1418825..1419823	-	332	WP_048902684.1	BN877_RS06880	hypothetical protein	-
1419778..1420464	-	228	WP_022556141.1	BN877_RS06885	hypothetical protein	-
1420464..1421165	-	233	WP_022556142.1	BN877_RS06890	hypothetical protein	-
1421169..1421606	-	145	WP_022556143.1	BN877_RS06895	hypothetical protein	-
1421669..1422130	-	153	WP_022556144.1	BN877_RS06900	hypothetical protein	-
1422203..1423300	-	365	WP_022556145.1	BN877_RS06905	N4-gp56 family major capsid protein	-
1423539..1424201	+	220	WP_052349963.1	BN877_RS06910	hypothetical protein	-
1424722..1425054	+	110	WP_022556148.1	BN877_RS06920	conserved membrane protein of unknown	-
1425259..1425489	+	76	WP_022556149.1	BN877_RS06925	hypothetical protein	-
1545167..1546057	+	296	WP_022556225.1	BN877_RS07515	putative NAD dependent	COG0451M
1558362..1559330	-	322	WP_022556232.1	BN877_RS07580	Dienelactone hydrolase-like protein	COG4188R

1652597..1652875	+	92	WP_004442197.1	BN877_RS07970	hypothetical protein	COG2929S
1652865..1653197	+	110	WP_006698844.1	BN877_RS07975	transcriptional regulator	COG2944K
1715896..1716333	+	145	WP_022556324.1	BN877_RS08295	hypothetical protein	-
1716326..1716745	+	139	WP_022556325.1	BN877_RS08300	transcriptional regulator	-
1716751..1717254	+	167	WP_048902695.1	BN877_RS08305	hypothetical protein	-
1717640..1718824	-	394	WP_022556328.1	BN877_RS08315	hypothetical protein	COG0582LX
1718961..1719179	-	72	WP_022556329.1	BN877_RS08320	hypothetical protein	-
1719176..1719448	-	90	WP_022556330.1	BN877_RS08325	hypothetical protein	-
1719445..1719711	-	88	WP_022556331.1	BN877_RS08330	hypothetical protein	-
1719708..1720037	-	109	WP_022556332.1	BN877_RS08335	hypothetical protein	-
1720030..1720275	-	81	WP_022556333.1	BN877_RS08340	hypothetical protein	-
1720272..1720553	-	93	WP_048902696.1	BN877_RS08345	hypothetical protein	-
1720823..1721479	-	218	WP_048902697.1	BN877_RS08350	hypothetical protein	-
1721544..1721852	-	102	WP_048902698.1	BN877_RS08355	hypothetical protein	-
1722093..1722806	-	237	WP_048902700.1	BN877_RS08365	hypothetical protein	-
1722967..1723272	-	101	WP_048902701.1	BN877_RS08370	hypothetical protein	-
1723276..1723671	+	131	WP_022556340.1	BN877_RS08375	hypothetical protein	-
1723668..1723847	+	59	WP_022556341.1	BN877_RS08380	hypothetical protein	-
1723844..1724125	+	93	WP_022556342.1	BN877_RS08385	hypothetical protein	-
1724130..1724936	+	268	WP_022556343.1	BN877_RS08390	hypothetical protein	-
1724933..1725145	+	70	WP_048902702.1	BN877_RS08395	hypothetical protein	-
1725217..1725462	+	81	WP_022556344.1	BN877_RS08400	hypothetical protein	-
1725459..1725725	+	88	WP_022556345.1	BN877_RS08405	hypothetical protein	-
1725727..1725996	+	89	WP_022556346.1	BN877_RS08410	hypothetical protein	-
1725993..1727069	+	358	WP_022556347.1	BN877_RS08415	hypothetical protein	COG4643X
1727060..1727467	+	135	WP_048902703.1	BN877_RS08420	hypothetical protein	-
1727464..1728513	+	349	WP_048902704.1	BN877_RS08425	hypothetical protein	-
1728586..1730436	+	616	WP_022556350.1	BN877_RS08430	hypothetical protein	COG3378X
1730830..1731423	+	197	WP_022556352.1	BN877_RS08435	Holliday junction resolvase	-
1731938..1733611	+	557	WP_022556353.1	BN877_RS08440	putative TerL	-
1733972..1734196	+	74	WP_022556355.1	BN877_RS08445	hypothetical protein	-
1734196..1735734	+	512	WP_022556356.1	BN877_RS08450	hypothetical protein	-
1735736..1736101	+	121	WP_022556357.1	BN877_RS08455	putative ornithine aminotransferase RocD1	-
1736116..1736958	+	280	WP_022556358.1	BN877_RS08460	Multiubiquitin chain binding protein	-
1737109..1737978	+	289	WP_022556359.1	BN877_RS08465	hypothetical protein	-
1738040..1738318	+	92	WP_022556360.1	BN877_RS08470	hypothetical protein	-
1738387..1739016	+	209	WP_022556361.1	BN877_RS08475	hypothetical protein	-
1739037..1741046	+	669	WP_048902705.1	BN877_RS08480	hypothetical protein	-
1741060..1741626	+	188	WP_022556363.1	BN877_RS08485	putative golgin autoantigen	-
1741626..1744502	+	958	WP_022556364.1	BN877_RS08490	hypothetical protein	-
1744598..1746334	+	578	WP_048902706.1	BN877_RS08495	hypothetical protein	-
1746331..1748829	+	832	WP_022556366.1	BN877_RS08500	hypothetical protein	-
1748880..1750823	+	647	WP_022556367.1	BN877_RS08505	hypothetical protein	-
1750835..1751230	-	131	WP_048902707.1	BN877_RS08510	hypothetical protein	-
1751313..1752344	-	343	WP_022556369.1	BN877_RS08515	family 2 glycosyl transferase	COG1216G
1752667..1753563	+	298	WP_022556370.1	BN877_RS08520	putative transmembrane protein	COG4678MX
1753556..1753846	+	96	WP_022556371.1	BN877_RS08525	conserved exported protein of unknown	-
1753954..1754283	+	109	WP_022556372.1	BN877_RS08530	conserved membrane protein of unknown	-
1762481..1762714	-	77	WP_048902708.1	BN877_RS08595	hypothetical protein	-
1762989..1763189	-	66	WP_022556379.1	BN877_RS08605	hypothetical protein	-
1763914..1764279	-	121	WP_052349965.1	BN877_RS08615	hypothetical protein	COG1694V
1767055..1767255	+	66	WP_022556386.1	BN877_RS08630	dehydrogenase	-
1767245..1767721	+	158	WP_006697486.1	BN877_RS08635	hypothetical protein	-
1768571..1768984	+	137	WP_022556388.1	BN877_RS08645	hypothetical protein	-
1769680..1769952	+	90	WP_022556390.1	BN877_RS08655	hypothetical protein	-
1769999..1771003	+	334	WP_022556391.1	BN877_RS08660	exported protein of unknown function	-
1771142..1771804	+	220	WP_022556392.1	BN877_RS08665	hypothetical protein	COG3637M
1774560..1775201	+	213	WP_022556397.1	BN877_RS08690	hypothetical protein	-
1781994..1782638	-	214	WP_048902710.1	BN877_RS08725	hypothetical protein	-

1782638..1782988	-	116	WP_022556406.1	BN877_RS08730	hypothetical protein	-
1782990..1783178	+	62	WP_048902711.1	BN877_RS08735	hypothetical protein	-
1785578..1785913	+	111	WP_022556409.1	BN877_RS08745	conserved exported protein of unknown	-
1786091..1786801	+	236	WP_022556410.1	BN877_RS08750	hypothetical protein	-
1787084..1787443	-	119	WP_022556411.1	BN877_RS08755	hypothetical protein	-
1788812..1789168	-	118	WP_022556414.1	BN877_RS08775	phage head-tail adaptor	COG5614X
1789128..1789319	-	63	WP_048902712.1	BN877_RS08780	hypothetical protein	-
1796102..1796422	-	106	WP_048902713.1	BN877_RS08825	HNH endonuclease	COG1403V
1796483..1796905	+	140	WP_022556424.1	BN877_RS08830	hypothetical protein	-
1797214..1798158	-	314	WP_022556425.1	BN877_RS08840	hypothetical protein	-
1799037..1799984	-	315	WP_022556428.1	BN877_RS08850	hypothetical protein	-
1800214..1801596	-	460	WP_048902714.1	BN877_RS08855	hypothetical protein	COG2865K
1801662..1803884	-	740	WP_022556430.1	BN877_RS08860	RecA-family ATPase	-
1805160..1805393	-	77	WP_022556433.1	BN877_RS08875	hypothetical protein	-
1805390..1806448	-	352	WP_022556434.1	BN877_RS08880	DNA methylase N-4/N-6 domain protein	COG0863L
1806448..1807851	-	467	WP_048902834.1	BN877_RS08885	helicase	COG0553KL
1807938..1808708	+	256	WP_022556436.1	BN877_RS08890	hypothetical protein	-
1809412..1809861	+	149	WP_022556438.1	BN877_RS08900	hypothetical protein	-
1811708..1812199	+	163	WP_022556440.1	BN877_RS08910	hypothetical protein	-
1815730..1816308	-	192	WP_022556445.1	BN877_RS08935	hypothetical protein	-
1817697..1818008	-	103	WP_022556449.1	BN877_RS08950	hypothetical protein	-
1820444..1820650	-	68	WP_037092366.1	BN877_RS08975	hypothetical protein	-
1820940..1821575	+	211	WP_048902715.1	BN877_RS08980	hypothetical protein	-
1822515..1822766	+	83	WP_048902716.1	BN877_RS08990	hypothetical protein	-
1822799..1823011	+	70	WP_048902717.1	BN877_RS08995	hypothetical protein	-
1823011..1823466	+	151	WP_022556459.1	BN877_RS09000	hypothetical protein	-
1823456..1824046	+	196	WP_022556460.1	BN877_RS09005	hypothetical protein	-
1853775..1854014	+	79	WP_045533784.1	BN877_RS09150	hypothetical protein	-
1938516..1939691	-	391	WP_004442617.1	BN877_RS09635	elongation factor Tu	COG0050J
1962395..1962844	-	149	WP_004442652.1	BN877_RS09735	hypothetical protein	-
1986179..1987210	+	343	WP_022556555.1	BN877_RS09875	hypothetical protein	COG2374R
1991607..1992872	-	421	WP_004442710.1	BN877_RS09900	NADH dehydrogenase	COG1252C
2007739..2008488	-	249	WP_022556569.1	BN877_RS09985	SDR family oxidoreductase	COG1028IQR
2010332..2011219	-	295	WP_022556571.1	BN877_RS09995	conserved membrane protein of unknown	COG0697GER
2108549..2108731	-	60	WP_044459625.1	BN877_RS10420	hypothetical protein	-
2118614..2119168	+	184	WP_048902727.1	BN877_RS10470	hypothetical protein	-
2123199..2124458	+	419	WP_048902728.1	BN877_RS10480	mannose-6-phosphate isomerase	COG2942G
2138536..2138988	-	150	WP_048902729.1	BN877_RS10545	hypothetical protein	COG5628R
2152223..2152426	+	67	WP_006699640.1	BN877_RS10595	hypothetical protein	COG4877S
2301749..2302456	-	235	WP_004443119.1	BN877_RS11330	hypothetical protein	-
2343399..2343695	+	98	WP_006699572.1	BN877_RS11515	hypothetical protein	COG1669R
2343695..2344048	+	117	WP_006699571.1	BN877_RS11520	hypothetical protein	COG2361S
2384617..2385204	+	195	WP_022556814.1	BN877_RS11730	putative ATPase	COG3911R
2426037..2426231	-	64	WP_022556832.1	BN877_RS11950	hypothetical protein	-
2426228..2426788	-	186	WP_037091853.1	BN877_RS11955	hypothetical protein	-
2428961..2430028	+	355	WP_006700422.1	BN877_RS11970	iron ABC transporter	COG1840P
2443619..2444428	+	269	WP_004443469.1	BN877_RS12050	lipase	COG0657I
2444544..2445734	+	396	WP_004443470.1	BN877_RS12055	MexE family multidrug efflux RND transporter periplasmic adaptor subunit	COG0845MV
2620200..2620424	+	74	WP_048902754.1	BN877_RS12795	hypothetical protein	-
2666575..2667021	+	148	WP_048902755.1	BN877_RS13030	protein-tyrosine-phosphatase	COG0394T
2693559..2694356	+	265	WP_004443949.1	BN877_RS13170	amino acid ABC transporter	COG0834ET
2721569..2721889	-	106	WP_048902757.1	BN877_RS13280	hypothetical protein	-
2741221..2742381	+	386	WP_022556991.1	BN877_RS13330	hypothetical protein	COG3970R
2744255..2745262	+	335	WP_004444024.1	BN877_RS13345	ABC transporter substrate-binding protein	COG1638G
2779290..2779562	-	90	WP_048902760.1	BN877_RS13475	hypothetical protein	-
				**Linear chromosome**		
1399..1719	+	106	WP_022562567.1	BN877_RS13810	hypothetical protein	-
2866..3543	-	225	WP_035210577.1	BN877_RS13825	hypothetical protein	-

4844..5095	+	83	WP_048902861.1	BN877_RS13835	hypothetical protein	-
5348..5782	+	144	WP_048902949.1	BN877_RS13840	hypothetical protein	-
5972..6493	+	173	WP_022562575.1	BN877_RS13845	hypothetical protein	-
7967..8206	-	79	WP_022562576.1	BN877_RS13860	hypothetical protein	-
8536..9978	-	480	WP_048902862.1	BN877_RS13865	hypothetical protein	-
10371..11444	+	357	WP_022562578.1	BN877_RS13870	Zinc finger, CHC2-family protein	COG0358L
11441..14665	+	1074	WP_022562579.1	BN877_RS13875	hypothetical protein	COG3344X
14758..15570	+	270	WP_048902863.1	BN877_RS13880	hypothetical protein	COG0388R
15635..16504	+	289	WP_022562582.1	BN877_RS13885	DNA polymerase III subunit epsilon	COG0847L
17149..19185	+	678	WP_052349966.1	BN877_RS13890	hypothetical protein	COG1479S
19266..22199	-	977	WP_022562585.1	BN877_RS13895	DNA modification methyltransferase-related	COG1002V
22654..23139	+	161	WP_048902864.1	BN877_RS13900	hypothetical protein	-
72533..74044	-	503	WP_006700548.1	BN877_RS14140	alpha-N-arabinofuranosidase	COG3534G
80627..81403	+	258	WP_022562631.1	BN877_RS14170	hypothetical protein	COG1082G
81491..81751	+	86	WP_022562632.1	BN877_RS14175	hypothetical protein	-
81748..82227	+	159	WP_006700540.1	BN877_RS14180	hypothetical protein	-
95014..95256	+	80	WP_006700407.1	BN877_RS14255	hypothetical protein	-
116453..118456	-	667	WP_022562658.1	BN877_RS14365	oxidoreductase	COG0673R
133962..134972	-	336	WP_048902953.1	BN877_RS14445	zinc-binding dehydrogenase	COG1063ER
134989..135759	-	256	WP_022562672.1	BN877_RS14450	sorbitol dehydrogenase	COG1028IQR
135938..137251	-	437	WP_022562673.1	BN877_RS14455	putative ABC transporter, periplasmic	COG1653G
137563..138570	+	335	WP_048902872.1	BN877_RS14460	sugar kinase	COG0524G
138567..139844	+	425	WP_022562675.1	BN877_RS14465	D-tagatose-bisphosphate aldolase, class II,	COG4573G
142909..143985	-	358	WP_022562679.1	BN877_RS14485	ABC transporter ATP-binding protein	COG3842E
143990..144820	-	276	WP_022562680.1	BN877_RS14490	ABC transporter, membrane spanning	COG0395G
144824..145768	-	314	WP_003525091.1	BN877_RS14495	sugar ABC transporter permease	COG1175G
145920..147179	-	419	WP_022562681.1	BN877_RS14500	ABC transporter, substrate binding protein	COG1653G
147218..148390	-	390	WP_022562682.1	BN877_RS14505	hypothetical protein	COG0626E
148387..149079	-	230	WP_048902873.1	BN877_RS14510	GntR family transcriptional regulator	COG2186K
149310..149783	-	157	WP_048902874.1	BN877_RS14515	ribonuclease H	COG0328L
164342..165583	-	413	WP_006700373.1	BN877_RS14595	antibiotic transporter	COG2270R
268643..269059	+	138	WP_022562749.1	BN877_RS15055	hypothetical protein	-
388823..389116	-	97	WP_048902887.1	BN877_RS15565	hypothetical protein	-
390810..391157	+	115	WP_048902888.1	BN877_RS15575	hypothetical protein	-
391188..391619	-	143	WP_022562835.1	BN877_RS15580	putative RTX toxin-like gene	-
393214..394899	-	561	WP_022562839.1	BN877_RS15600	Choline dehydrogenase-like flavoprotein	COG2303IR
395049..395903	-	284	WP_022562840.1	BN877_RS15605	xylose isomerase	COG1082G
520944..521780	-	278	WP_022562938.1	BN877_RS16190	non-heme chloroperoxidase (Chloride peroxidase) (CPO-P) (Chloroperoxidase P)	COG2267I
555107..555325	-	72	WP_048902892.1	BN877_RS16355	hypothetical protein	-
564073..565188	+	371	WP_048902893.1	BN877_RS16395	acyltransferase	COG1835M
632533..633063	-	176	WP_048902897.1	BN877_RS16720	hypothetical protein	-
661198..661398	-	66	WP_048902899.1	BN877_RS16850	hypothetical protein	-
761223..761312	-	29	WP_003506089.1	BN877_RS17295	ATPase	-
766413..766592	-	59	WP_034497343.1	BN877_RS17320	hypothetical protein	-
772786..773820	-	344	WP_022563088.1	BN877_RS17345	transcriptional regulator	COG1609K
813049..813465	-	138	WP_022563116.1	BN877_RS17535	hypothetical protein	-
838216..839901	+	561	WP_022563137.1	BN877_RS17640	aspartate-alanine antiporter	COG2985R
839920..841509	+	529	WP_004445962.1	BN877_RS17645	aspartate 4-decarboxylase	COG0436E
853722..854045	-	107	WP_022563150.1	BN877_RS17705	hypothetical protein	-
878156..878554	+	132	WP_022563170.1	BN877_RS17815	hypothetical protein	-
878572..879225	+	217	WP_004446016.1	BN877_RS17820	transcriptional regulator	COG1309K
879284..882202	-	972	WP_048902908.1	BN877_RS17825	adhesin	COG3468MU
883906..884367	-	153	WP_022563174.1	BN877_RS17835	hypothetical protein	COG3918S
884676..885416	-	246	WP_022563175.1	BN877_RS17840	siderophore-iron reductase FhuF	COG4114P
885430..886254	-	274	WP_048902971.1	BN877_RS17845	iron compound ABC transporter substrate-	-
886299..888494	-	731	WP_022563177.1	BN877_RS17850	TonB-dependent receptor	COG4774P
888780..889526	+	248	WP_022563178.1	BN877_RS17855	AraC family transcriptional regulator	COG2207K
889523..890380	+	285	WP_022563179.1	BN877_RS17860	cobalamin ABC transporter ATPase	COG1120PH

890377..892353	+	658	WP_022563180.1	BN877_RS17865	ABC-type Fe3+-siderophore transport	COG0609P
892465..893325	-	286	WP_022563181.1	BN877_RS17870	hydrolase or acyltransferase of alpha/beta	COG0596HR
893435..894355	-	306	WP_034497688.1	BN877_RS17875	secretion protein HlyD	COG1566V
894358..894564	-	68	WP_022563183.1	BN877_RS17880	hypothetical protein	-
894561..896564	-	667	WP_022563184.1	BN877_RS17885	hypothetical protein	COG1289S
896561..896932	-	123	WP_022563185.1	BN877_RS17890	hypothetical protein	-
897006..897812	+	268	WP_022563186.1	BN877_RS17895	transcriptional regulator	COG2207K
897852..898226	+	124	WP_022563187.1	BN877_RS17900	lactoylglutathione lyase-like lyase	COG0346Q
898291..899187	-	298	WP_022563188.1	BN877_RS17905	LysR family transcriptional regulator	COG0583K
899311..900282	+	323	WP_022563189.1	BN877_RS17910	Zn-dependent hydrolase, glyoxylase	COG0491R
900316..900900	+	194	WP_022563190.1	BN877_RS17915	hypothetical protein	COG3576R
900989..902080	-	363	WP_022563191.1	BN877_RS17920	arabinose efflux permease family protein	COG2814G
902148..902504	-	118	WP_022563192.1	BN877_RS17925	antibiotic biosynthesis monooxygenase	-
902629..903555	+	308	WP_022563194.1	BN877_RS17930	LysR family transcriptional regulator	COG0583K
903707..904657	+	316	WP_022563195.1	BN877_RS17935	transcriptional regulator	COG0583K
904679..905758	-	359	WP_022563196.1	BN877_RS17940	hydrolase	COG1680V
911918..913297	+	459	WP_022563203.1	BN877_RS17970	hypothetical protein	-
913524..914633	+	369	WP_006697873.1	BN877_RS17975	signal transduction histidine kinase	COG3920T
914645..915070	+	141	WP_004446066.1	BN877_RS17980	transcriptional regulator	COG0784T
915097..917406	+	769	WP_022563204.1	BN877_RS17985	PAS domain-containing protein	-
918619..918924	-	101	WP_034497513.1	BN877_RS17990	hypothetical protein	-
919093..919488	+	131	WP_004446075.1	BN877_RS17995	membrane protein	-
919673..921043	+	456	WP_022563206.1	BN877_RS18000	signal transduction histidine kinase	COG4585T
921040..921687	+	215	WP_022563207.1	BN877_RS18005	DNA-binding response regulator	COG2197TK
921795..922352	+	185	WP_022563208.1	BN877_RS18010	hypothetical protein	-
925979..926176	+	65	WP_004446090.1	BN877_RS18035	hypothetical protein	-
926950..927855	-	301	WP_004446094.1	BN877_RS18045	LysR family transcriptional regulator	COG0583K
927966..928715	+	249	WP_022563213.1	BN877_RS18050	short-chain dehydrogenase	COG1028IQR
929228..931183	+	651	WP_022563214.1	BN877_RS18055	methyl-accepting chemotaxis protein	COG2770T
931333..932550	-	405	WP_022563215.1	BN877_RS18060	diguanylate cyclase	COG3706TK
932999..933496	+	165	WP_022563216.1	BN877_RS18065	hypothetical protein	-
933848..934327	-	159	WP_022563217.1	BN877_RS18070	choline dehydrogenase	-
934327..935118	-	263	WP_022563218.1	BN877_RS18075	hydrolase	COG0596HR
935263..935730	+	155	WP_022563219.1	BN877_RS18080	Organic hydroperoxide resistance	COG1846K
935760..936539	-	259	WP_022563220.1	BN877_RS18085	short-chain dehydrogenase	COG1028IQR
936536..936862	-	108	WP_022563221.1	BN877_RS18090	hypothetical protein	COG3631R
936999..937895	+	298	WP_022563222.1	BN877_RS18095	transcriptional regulator	COG0583K
938112..939092	-	326	WP_022563223.1	BN877_RS18100	glycerate dehydrogenase	COG1052CHR
939161..940648	-	495	WP_034497708.1	BN877_RS18105	hypothetical protein	COG3333R
940681..941145	-	154	WP_022563224.1	BN877_RS18110	hypothetical protein	-
941145..942101	-	318	WP_022563225.1	BN877_RS18115	hypothetical protein	COG3181C
942196..942879	-	227	WP_022563226.1	BN877_RS18120	demethylmenaquinone methyltransferase	COG0684J
942912..943904	-	330	WP_022563227.1	BN877_RS18125	D-3-phosphoglycerate dehydrogenase	COG0111HR
944084..944965	+	293	WP_022563228.1	BN877_RS18130	transcriptional regulator	COG0583K
945867..946577	+	236	WP_022563230.1	BN877_RS18135	GntR family transcriptional regulator	COG1802K
946612..947727	+	371	WP_004446116.1	BN877_RS18140	sn-glycerol-3-phosphate ABC transporter	COG3842E
947724..948653	+	309	WP_022563231.1	BN877_RS18145	sugar ABC transporter permease	COG4209G
948664..949545	+	293	WP_006697906.1	BN877_RS18150	sugar ABC transporter permease	COG0395G
949606..951135	+	509	WP_034497518.1	BN877_RS18155	sugar ABC transporter permease	COG1653G
951206..953536	+	776	WP_022563233.1	BN877_RS18160	oligo alginate lyase	-
953556..954305	+	249	WP_022563234.1	BN877_RS18165	SDR family oxidoreductase	COG1028IQR
954321..955490	+	389	WP_022563235.1	BN877_RS18170	lysophospholipase L1-like esterase	COG2755E
963116..963334	-	72	WP_022563243.1	BN877_RS18205	MarR family transcriptional regulator	-
964277..964942	-	221	WP_022563245.1	BN877_RS18215	nucleoside triphosphate hydrolase	COG0572F
964969..965817	-	282	WP_022563246.1	BN877_RS18220	tagatose 3-epimerase	COG1082G
965819..966814	-	331	WP_022563247.1	BN877_RS18225	sugar ABC transporter permease	COG1172G
966814..967827	-	337	WP_006697919.1	BN877_RS18230	sugar ABC transporter permease	COG1172G
967820..969310	-	496	WP_022563248.1	BN877_RS18235	putative sugar ABC transporter, ATP-	COG1129G
969427..970404	-	325	WP_003521644.1	BN877_RS18240	sugar ABC transporter substrate-binding	COG1879G

970642..971721	+	359	WP_022563250.1	BN877_RS18245	LacI family transcriptional regulator	COG1609K
971731..972060	+	109	WP_034497523.1	BN877_RS18250	hypothetical protein	-
972562..972960	-	132	WP_022563252.1	BN877_RS18255	hypothetical protein	-
1035171..1036241	+	356	WP_022563294.1	BN877_RS18525	ATP-binding protein of sugar ABC	COG3839G
1109489..1110088	+	199	WP_022563348.1	BN877_RS18825	hypothetical protein	-
1174200..1175450	-	416	WP_022563403.1	BN877_RS19145	adenylate cyclase	COG2114T
1236473..1237516	-	347	WP_022563438.1	BN877_RS19425	hypothetical protein	COG4427S
1256080..1256259	+	59	WP_048902913.1	BN877_RS19500	hypothetical protein	-
1312464..1313255	+	263	WP_001206316.1	BN877_RS19775	ANT(3\'\')-Ia family aminoglycoside	COG1708R
1364722..1365150	+	142	WP_044459934.1	BN877_RS19985	acetyl-CoA carboxylase biotin carboxyl	COG0511HI
1365150..1366532	+	460	WP_022563501.1	BN877_RS19990	acetyl-CoA carboxylase biotin carboxylase	COG0439I
1366529..1366945	+	138	WP_022563502.1	BN877_RS19995	hypothetical protein	COG0511HI
1366942..1367649	+	235	WP_022563503.1	BN877_RS20000	putative hydrolase subunit	COG2049E
1367646..1368641	+	331	WP_022563504.1	BN877_RS20005	Biotin-dependent carboxylase-like protein	COG1984E
1368661..1369428	+	255	WP_004438027.1	BN877_RS20010	lactam utilization protein B	COG1540R
1369477..1370682	+	401	WP_004438028.1	BN877_RS20015	aspartate aminotransferase	COG0436E
1370727..1371632	-	301	WP_004438029.1	BN877_RS20020	LysR family transcriptional regulator	COG0583K
1371860..1372639	+	259	WP_022563506.1	BN877_RS20025	short-chain alcohol dehydrogenase	COG4221C
1372636..1373343	+	235	WP_004438031.1	BN877_RS20030	ABC transporter permease	COG0765E
1373340..1374098	+	252	WP_022563507.1	BN877_RS20035	Amine acid ABC transporter, permease protein, 3-TM region, His/Glu/Gln/Arg/opine	COG0765E
1374095..1374856	+	253	WP_022563508.1	BN877_RS20040	amino acid transporter ATP-binding protein	COG1126E
1374888..1375724	+	278	WP_022563509.1	BN877_RS20045	Periplasmic component of amino acid ABC-type transporter/signal transduction system	COG0834ET
1375824..1376498	+	224	WP_004438040.1	BN877_RS20050	S-adenosylmethionine--2-	COG0684J
1553645..1553869	+	74	WP_035208549.1	BN877_RS20810	hypothetical protein	-
1597127..1597357	+	76	WP_048902924.1	BN877_RS21025	hypothetical protein	-
1628681..1629847	-	388	WP_022563676.1	BN877_RS21150	alanine racemase	COG0787M
1682183..1682371	-	62	WP_022563705.1	BN877_RS21375	hypothetical protein	-
1690617..1691567	-	316	WP_022563711.1	BN877_RS21415	Nucleotidyltransferase protein (modular	COG2250S
1691564..1692217	-	217	WP_022563712.1	BN877_RS21420	hypothetical protein	-
1692404..1692607	+	67	WP_006697238.1	BN877_RS21425	transcriptional regulator	COG1476K
1725954..1727003	-	349	WP_022563731.1	BN877_RS21570	sugar ABC transporter permease	COG0395G
1727006..1728001	-	331	WP_022563732.1	BN877_RS21575	putative sugar ABC transporter, permease	COG1175G
1728003..1729106	-	367	WP_022563733.1	BN877_RS21580	sugar ABC transporter ATP-binding protein	COG3842E
1729106..1730458	-	450	WP_048902986.1	BN877_RS21585	polygalacturonase	COG5434G
1730464..1731558	-	364	WP_022563735.1	BN877_RS21590	putative unsaturated glucuronyl hydrolase	COG4225G
1731572..1734094	-	840	WP_022563736.1	BN877_RS21595	hypothetical protein	-
1734290..1735570	+	426	WP_022563737.1	BN877_RS21600	ABC-type sugar transport system,	COG1653G
1735776..1737758	+	660	WP_022563738.1	BN877_RS21605	serine protease	COG4625S
1737815..1739038	-	407	WP_022563739.1	BN877_RS21610	major facilitator superfamily protein	COG2223P
1739038..1741467	-	809	WP_022563740.1	BN877_RS21615	Ferrichrome-iron transporter	COG4773P
1741577..1742545	-	322	WP_022563741.1	BN877_RS21620	FecR iron sensor protein	COG3712PT
1742630..1743136	-	168	WP_006697272.1	BN877_RS21625	RNA polymerase sigma factor	COG1595K
1743331..1744479	-	382	WP_048902929.1	BN877_RS21630	NagC family transcriptional regulator	COG1940KG
1744689..1745585	+	298	WP_022563744.1	BN877_RS21635	glycerophosphoryl diester phosphodiesterase	COG0584I
1745717..1746466	-	249	WP_022563745.1	BN877_RS21640	short-chain dehydrogenase/reductase	COG1028IQR
1746562..1747470	+	302	WP_022563746.1	BN877_RS21645	DNA-binding domain-containing protein,	COG2207K
1747627..1748760	-	377	WP_022563747.1	BN877_RS21650	Transcriptional regulator/sugar kinase	COG1940KG
1749049..1750041	+	330	WP_022563748.1	BN877_RS21655	putative ABC transporter, substrate-binding	COG1840P
1750153..1751238	+	361	WP_022563749.1	BN877_RS21660	iron ABC transporter ATP-binding protein	COG3842E
1751235..1752920	+	561	WP_022563750.1	BN877_RS21665	ABC transporter permease	COG1178P
1753001..1753957	+	318	WP_022563751.1	BN877_RS21670	hypothetical protein	-
1754305..1755129	+	274	WP_022563753.1	BN877_RS21675	transposase	COG3547X
1755132..1755701	-	189	WP_022563754.1	BN877_RS21680	hypothetical protein	COG1853C
1755713..1756048	-	111	WP_004438973.1	BN877_RS21685	4Fe-4S ferredoxin	COG1146F
1756152..1756751	-	199	WP_048902987.1	BN877_RS21690	superoxide dismutase	COG0605P
1756943..1761370	-	1475	WP_048902988.1	BN877_RS21695	hemagglutinin	-
1761606..1762175	-	189	WP_022563757.1	BN877_RS21700	TetR family transcriptional regulator	COG1309K

1762223..1762750	+	175	WP_022563758.1	BN877_RS21705	dihydrofolate reductase	COG0262H
1762791..1763858	-	355	WP_022563759.1	BN877_RS21710	choloylglycine hydrolase	COG3049MR
1763999..1764799	+	266	WP_048902930.1	BN877_RS21715	hypothetical protein	COG2067I
1772593..1773543	+	316	WP_022563766.1	BN877_RS21740	Uncharacterized sodium-dependent	COG0385R
1773592..1774647	-	351	WP_022563767.1	BN877_RS21745	putative oxidoreductase	COG0667R
1774831..1775736	+	301	WP_022563768.1	BN877_RS21750	transcriptional regulator	COG0583K
1775817..1776602	-	261	WP_022563769.1	BN877_RS21755	hypothetical protein	COG3971Q
1779603..1780673	+	356	WP_048902931.1	BN877_RS21765	hypothetical protein	-
1780679..1781860	+	393	WP_048902932.1	BN877_RS21770	hypothetical protein	-
1781862..1782959	+	365	WP_022563774.1	BN877_RS25860	hypothetical protein	-
1782981..1783808	+	275	WP_022563775.1	BN877_RS21780	hypothetical protein	-
1783819..1784658	+	279	WP_022563776.1	BN877_RS21785	hypothetical protein	-
1784756..1785895	-	379	WP_022563777.1	BN877_RS21790	Lipopolysaccharide biosynthesis protein-like	COG3754M
1793031..1793351	+	106	WP_048902934.1	BN877_RS21820	hypothetical protein	-
1801188..1801925	-	245	WP_022563792.1	BN877_RS21860	dipeptide ABC transporter, nucleotide	COG1124EP
1802754..1803653	-	299	WP_022563794.1	BN877_RS21870	dipeptide ABC transporter, permease	COG1173EP
1851389..1852300	+	303	WP_022563834.1	BN877_RS22085	DMT(drug/metabolite transporter)	COG0697GER
1894358..1895923	+	521	WP_006700310.1	BN877_RS22260	peptide ABC transporter	COG0747E
1896146..1897114	+	322	WP_004444583.1	BN877_RS22265	ABC transporter permease	COG0601EP
1897133..1898080	+	315	WP_006700311.1	BN877_RS22270	ABC transporter permease	COG1173EP
1898086..1899279	+	397	WP_048902938.1	BN877_RS22275	cytosine deaminase	COG0402FR
1899279..1900772	+	497	WP_022563862.1	BN877_RS22280	Cytosine deaminase-like metal-dependent	COG0402FR
1900765..1901748	+	327	WP_004444579.1	BN877_RS22285	peptide ABC transporter substrate-binding	COG0444EP
1901745..1902767	+	340	WP_022563863.1	BN877_RS22290	putative ATP-binding protein of oligopeptide	COG4608E
1934993..1935808	-	271	WP_034498486.1	BN877_RS22430	ABC transporter permease	COG1177E
1956600..1956791	+	63	WP_022563909.1	BN877_RS22555	hypothetical protein	-
1957820..1958344	+	174	WP_022563912.1	BN877_RS22570	DNA-directed RNA polymerase sigma-70	COG1595K
1968303..1969337	-	344	WP_022563925.1	BN877_RS22645	putative sugar-nucleotide	COG0451M
1969312..1970463	-	383	WP_022563926.1	BN877_RS22650	conserved hypothetical protein; putative signal peptide; putative glycosyltransferase	COG4641D
1970460..1971551	-	363	WP_022563927.1	BN877_RS22655	hypothetical protein	COG4641D
1971548..1972678	-	376	WP_022563928.1	BN877_RS22660	conserved hypothetical protein; putative	COG4641D
1972682..1973797	-	371	WP_022563929.1	BN877_RS22665	glycosyl transferase group 1	COG0438M
1973794..1975830	-	678	WP_022563930.1	BN877_RS22670	putative nucleoside diphosphate epimerase	COG0451M
1975827..1976933	-	368	WP_022563931.1	BN877_RS22675	putative NAD-dependent epimerase/dehydratase; putative UDP-	COG0451M
1980514..1980843	-	109	WP_048902943.1	BN877_RS22710	hypothetical protein	-
1991516..1991980	-	154	WP_022563945.1	BN877_RS22760	conserved exported protein of unknown	-
1992255..1992629	-	124	WP_004444449.1	BN877_RS22765	two-component system response regulator	COG4566TK
1992696..1993352	-	218	WP_006700524.1	BN877_RS22770	DNA-binding response regulator	COG4566TK
1995124..1997418	-	764	WP_022563947.1	BN877_RS22780	putative formate dehydrogenase (C-terminal), related to acid resistance with formate dehydrogenase/DMSO reductase,	COG0243C
1998365..1999345	+	326	WP_022563948.1	BN877_RS22790	Non-heme chloroperoxidase (Chloride peroxidase) (CPO-P) (Chloroperoxidase P)	COG2267I
1999411..1999608	+	65	WP_048902944.1	BN877_RS22795	hypothetical protein	-
1999739..1999945	+	68	WP_022563949.1	BN877_RS22800	hypothetical protein	-
2000063..2001385	-	440	WP_022563950.1	BN877_RS22805	putative epoxide hydrolase	COG0596HR
2001566..2002984	-	472	WP_048902945.1	BN877_RS22810	aldehyde dehydrogenase	COG1012C
2003275..2003691	-	138	WP_022563952.1	BN877_RS22815	organic hydroperoxide resistance protein	COG1764V
2003835..2005151	-	438	WP_022563953.1	BN877_RS22820	two-component sensor histidine kinase	COG0642T
2005148..2005888	-	246	WP_022563954.1	BN877_RS22825	DNA-binding response regulator	COG0745TK
2005983..2007029	-	348	WP_022563955.1	BN877_RS22830	alpha/beta hydrolase	COG0596HR
2007234..2007992	-	252	WP_022563956.1	BN877_RS22835	hypothetical protein	COG0596HR
2008029..2009807	-	592	WP_022563957.1	BN877_RS22840	cytochrome C biogenesis protein DipZ	COG0785CO
2027472..2027792	-	106	WP_022563979.1	BN877_RS22930	hypothetical protein	-
2029797..2030294	-	165	WP_022563983.1	BN877_RS22945	hypothetical protein	COG5654S
2032772..2033602	+	276	WP_022563986.1	BN877_RS22960	hypothetical protein	COG5340V
2033589..2034509	+	306	WP_022563987.1	BN877_RS22965	hypothetical protein	COG2253V

2034525..2034887	+	120	WP_048902947.1	BN877_RS22970	hypothetical protein	-
2034901..2035185	-	94	WP_022563989.1	BN877_RS22975	hypothetical protein	-
