# Supplementary material for: Genomic Comparison of Agrobacterium pusense Strains Isolated from Bean Nodules
Source: Front Microbiol. 2016 Oct 27;7:1720. doi: 10.3389/fmicb.2016.01720 (PMC5081363; doi:10.3389/fmicb.2016.01720)
Supplement: Supplementary file 3 [file Table3.PDF]

Supplementary table 3. COG functional classification of genes of strains CCGM10 and CCGM11.

	Class	CCGM10								CCGM11							
	INFORMATION STORAGE AND PROCESSING	ChrC	ChrL	plasmids	pE	pD	pC	pB	pA	ChrC	ChrL	plasmids	pE	pD	pC	pB	pA
J	Translation, ribosomal structure and biogenesis	194	29	6	1	4	0	1	0	188	30	4	1	3	0	-	0
K	Transcription	158	148	51	22	15	10	2	2	147	145	46	21	14	9	-	2
L	Replication, recombination and repair	93	24	42	13	18	5	4	2	86	23	34	14	13	5	-	2
B	Chromatin structure and dynamics	1	0	0	0	0	0	0	0	1	0	0	0	0	0	-	0
		446	201	99	36	37	15	7	4	422	198	84	36	30	14	-	4
	CELLULAR PROCESSES AND SIGNALING																
D	Cell cycle control, cell division, chromosome partitioning	21	13	18	9	4	2	2	1	19	12	17	9	4	3	-	1
V	Defense mechanisms	45	21	27	7	10	10	0	0	41	22	23	7	8	8	-	0
T	Signal transduction mechanisms	120	61	32	18	11	2	0	1	111	59	28	15	10	2	-	1
M	Cell wall/membrane/envelope biogenesis	145	82	29	12	7	10	0	0	135	80	23	7	6	10	-	0
N	Cell motility	48	15	12	3	6	1	0	2	45	13	10	2	6	0	-	2
W	Extracellular structures	2	0	0	0	0	0	0	0	2	0	0	0	0	0	-	0
U	Intracellular trafficking, secretion, and vesicular transport	27	18	45	7	21	17	0	0	26	17	40	8	17	15	-	0
O	Posttranslational modification, protein turnover, chaperones	107	31	25	12	6	6	1	0	104	27	23	11	6	6	-	0
		515	241	188	68	65	48	3	4	483	230	164	59	57	44	-	4
	METABOLISM																
C	Energy production and conversion	132	86	30	15	8	7	0	0	128	80	34	16	8	10	-	0
G	Carbohydrate transport and metabolism	141	209	18	7	7	1	0	3	135	199	17	6	7	1	-	3
E	Amino acid transport and metabolism	203	277	38	23	8	6	0	1	190	263	33	18	8	6	-	1
F	Nucleotide transport and metabolism	80	19	2	0	1	0	0	1	72	19	2	0	1	0	-	1
H	Coenzyme transport and metabolism	109	65	9	6	3	0	0	0	105	56	11	7	4	0	-	0
I	Lipid transport and metabolism	92	70	10	3	5	2	0	0	86	69	10	3	4	3	-	0
P	Inorganic ion transport and metabolism	126	91	41	17	12	11	0	1	117	83	38	16	9	12	-	1
Q	Secondary metabolites biosynthesis, transport and catabolism	37	40	4	2	2	0	0	0	35	36	4	2	2	0	-	0
		920	857	152	73	46	27	0	6	868	805	149	68	43	32	-	6
X	Prophages, transposons	28	22	68	17	28	7	14	2	26	17	57	18	29	8	-	2
	POORLY CHARACTERIZED																
R	General function prediction only	159	113	29	12	12	3	1	1	151	102	32	14	13	4	-	1
S	Function unknown	194	78	28	8	11	8	0	1	185	72	24	7	8	8	-	1
-	Not in COG	672	443	279	92	105	47	31	4	788	525	260	94	117	45	-	4
		1025	634	336	112	128	58	32	6	1124	699	316	115	138	57	-	6
	TOTAL	2934	1955	843	306	304	155	56	22	2923	1949	770	296	297	155	-	22

In the total lack unassigned genes, 55 for CCGM10 and 51 for CCGM11.
