# Supplementary material for: Genomic Comparison of Agrobacterium pusense Strains Isolated from Bean Nodules
Source: Front Microbiol. 2016 Oct 27;7:1720. doi: 10.3389/fmicb.2016.01720 (PMC5081363; doi:10.3389/fmicb.2016.01720)
Supplement: Supplementary file 4 [file Table4.PDF]

Supplementary table 4. Intact prophages detected in the genomes of *A. pusense* and *A. fabrum* strains.

	A. pusense				*A. fabrum*
Replicon	CCGM10	CCGM11	IRBG74	HPC(L)	C58
Circular chromosome	**Phage *RR1_A***<br>Size: 41.5 Kb<br>Coding genes: 54<br>Homologs: 24<br>GC: 57.9%<br><br>**Phage *Paraco_vB_IMEP1***<br>Size: 18.9 Kb<br>Coding genes: 21<br>Homologs: 4<br>GC: 62.9%<br><br>**Phage *Rhizob_16_3***<br>Size: 94.1 Kb<br>Coding genes: 94<br>Homologs: 28<br>GC: 57.3%	**Phage *RR1_A***<br>Size: 42.8 Kb<br>Coding genes: 57<br>Homologs: 23<br>GC: 59.4%<br><br>**Phage *Paraco_vB_IMEP1***<br>Size: 20.5 Kb<br>Coding genes: 22<br>Homologs: 4<br>GC: 65.8%<br><br>**Phage *Rhizob_16_3***<br>Size: 94.5 Kb<br>Coding genes: 98<br>Homologs: 27<br>GC: 57.5%	**Phage *Rhizob_16_3***<br>Size: 51.4 Kb<br>Coding genes: 64<br>Homologs: 21<br>GC: 57.3%	**Phage *Burkho_phi1026b***<br>Size: 17.3 Kb<br>Coding genes: 22<br>Homologs: 4<br>GC: 58.7%<br><br>**Phage *Paraco_vB_IMEP1***<br>Size: 16.6 Kb<br>Coding genes: 21<br>Homologs: 4<br>GC: 63.8%	**Phage *Aurant_AmM_1***<br>Size: 30.2 Kb<br>Coding genes: 41<br>Homologs: 7<br>GC: 57.1%<br><br>**Phage *Paraco_vB_IMEP1***<br>Size: 17.4 Kb<br>Coding genes: 21<br>Homologs: 4<br>GC: 64.1%
Linear chromosome	**Phage *Rhodob_RC1***<br>Size: 43.6 Kb<br>Coding genes: 48<br>Homologs: 15<br>GC: 59.5%	**Phage *Rhodob_RC1***<br>Size: 38.2 Kb<br>Coding genes: 53<br>Homologs: 16<br>GC: 60.6%			
Plasmid B	**Phage *Aurant_AmM_1***<br>Size: 37.2 Kb<br>Coding genes:47<br>Homologs: 12<br>GC: 58.2%				
